# Angiotensin II and angiotensin converting enzyme: key players in the pathogenesis of hypertensive retinopathy

**DOI:** 10.22336/rjo.2025.62

**Published:** 2025

**Authors:** Ecaterina Pavlovschi, Valeriana Pantea, Djina Borovic, Olga Tagadiuc

**Affiliations:** 1 Department of Biochemistry and Clinical Biochemistry, “Nicolae Testemițanu” State University of Medicine and Pharmacy, Chișinău, Republic of Moldova; 2 Laboratory of Biochemistry, “Nicolae Testemițanu” State University of Medicine and Pharmacy, Chișinău, Republic of Moldova; 3 Ovisus Medical Private Center, Chișinău, Republic of Moldova

**Keywords:** renin-angiotensin system, angiotensin II, angiotensin-converting enzyme, hypertensive retinopathy, HR = hypertensive retinopathy, HTN = hypertension, RAS = renin-angiotensin system, Ang II = angiotensin II, ACE = angiotensin-converting enzyme, KWB = Keith-Wagener-Barker

## Abstract

**Objective:**

To investigate the association between hypertensive retinopathy (HR) and components of the renin-angiotensin system (RAS), specifically angiotensin II (Ang II) and angiotensin-converting enzyme (ACE), as potential biomarkers for the diagnosis and prognosis of HR.

**Methods:**

A total of 90 patients diagnosed primarily with hypertension were prospectively enrolled. HR was graded according to the Keith-Wagener-Barker classification into three severity groups. Paired serum and tear fluid samples were collected from each participant to measure Ang II and ACE levels, assessing both systemic and ocular changes. Statistical analyses included tests for normality and variance, as well as appropriate non-parametric methods. A p-value <0.05 was considered significant.

**Results:**

Serum Ang II levels increased significantly with advancing stages of HR (p=0.039), showing a 42% rise in moderate HR compared to mild HR and an additional 18% increase in severe HR. Tear Ang II levels decreased markedly from mild to moderate HR (p=0.022) and from mild to severe HR (p=0.028). Serum ACE levels rose significantly as HR progressed (p=0.032), notably between mild and moderate HR (66% increase, p=0.009), whereas tear ACE levels showed no significant differences.

**Discussion:**

Our findings showed that serum Ang II and ACE levels rise with HR severity, indicating systemic RAS activation, while tear Ang II decreases, suggesting altered local regulation. These contrasting trends reflect tissue-specific RAS activity and support the potential of Ang II as a biomarker for HR progression. The unchanged tear ACE levels imply limited ocular surface involvement. Overall, the data underscore RAS components’ diagnostic relevance and therapeutic potential in hypertensive retinopathy.

**Conclusion:**

These findings suggest that Ang II levels in serum (increasing) and tears (decreasing) correlate with HR severity, while ACE changes are significant only in serum. Further studies are warranted to elucidate the role of RAS in HR pathophysiology and to explore its potential as a therapeutic target in ocular disease.

## Introduction

Hypertension (HTN) affects an estimated 1.28 billion adults worldwide aged 30 to 79, with two-thirds of cases occurring in low- and middle-income countries. Approximately 46% of adults with HTN remain undiagnosed, 42% receive therapy, and only around 21% achieve adequate blood pressure control [1,2]. This condition, often termed the “silent killer”, can damage various organs, including the eye. Hypertensive retinopathy (HR), the most frequent ocular manifestation of HTN, involves structural and metabolic changes in the retina [3,4]. Early stages of HR may be asymptomatic, and the diagnosis is often based on clinical findings such as narrowing of retinal arterioles, hemorrhages, exudates, or swelling of the optic disc [3,5].

Although high blood pressure is a key driver of HR, additional mechanisms - such as inflammation, angiogenesis, dyslipidemia, endothelial dysfunction, and oxidative stress (OS) - contribute to retinal vascular damage [6-12]. This is further supported by the observation that signs of HR can be present in individuals without a known history of HTN [13].

Early HR can be asymptomatic, making timely detection challenging, particularly when other ocular disorders (e.g., diabetic retinopathy (DR), age-related macular degeneration (AMD)) coexist [14]. Classification systems such as the Keith-Wagener-Barker (KWB), Scheie, and the modified Scheie are designed to standardize the evaluation of HR by assessing arteriolar and venular changes, retinal hemorrhages, and optic disc abnormalities, thereby enhancing consistency and communication among clinicians [15]. In recent years, scientists have been working to identify and establish novel reference points to standardize parameters and new diagnostic markers that can aid in the staging and prognosis of HR.

Given the above, the assessment of HR heavily relies on clinical examination, which necessitates the expertise of ophthalmologists and the use of specialized equipment. However, the interpretation of retinal findings may exhibit variability, potentially affecting the accuracy of the diagnostic process [3,16]. This is the critical juncture where markers can truly showcase their value and bring forth essential outcomes.

Biomarkers such as catalase, glutathione peroxidase, glutathione reductase, gamma-glutamyl transferase, von Willebrand factor, C-reactive protein, and various inflammatory cytokines have shown potential in reflecting the underlying pathological processes and providing additional diagnostic and prognostic information [8,9,17-21]. However, more research is needed to validate their utility and establish standardized protocols for their clinical implementation.

Our understanding of the renin-angiotensin system (RAS) has undergone significant advancements in recent years. Initially viewed as a classical endocrine system responsible for maintaining homeostasis by regulating circulating intravascular volume and restoring arterial pressure, it has evolved into a more comprehensive concept. We now recognize that the RAS consists of multiple local systems operating independently within different organs, including the eye [22,23].

It is known that RAS plays a critical role in regulating blood pressure and is intimately linked to the development of HTN, and possibly HR.

In HTN, there is often dysregulation within the RAS, characterized by an overproduction of Ang II or increased sensitivity to its effects, that contributes to vasoconstriction, increased vascular resistance, and sodium and fluid retention. Targeting RAS has been a key strategy in the management of HTN [12,24].

Recently, the hypothesis that a local RAS would be involved in the development and progression of HR, contributing to the pathological changes observed in the retinal vasculature, has been discussed more and more frequently.

Ang II, the key component of RAS, besides its vasoconstrictive effect, promotes inflammation, OS, and abnormal angiogenesis, all of which are considered to be involved in the development of HR. In addition, elevated levels of Ang II can lead to increased vascular permeability, leakage, and the formation of microaneurysms in the retinal blood vessels [12]. Furthermore, RAS activation can contribute to endothelial dysfunction, impaired blood flow regulation, and increased production of pro-inflammatory cytokines in the retina. These processes play a role in the progression of HR [25].

The identification and modulation of RAS components and their effects must be more intensively explored as potential targets for the management of HR. Understanding the interplay between RAS and HR can provide insights into potential therapeutic approaches for managing the condition and highlight the importance of targeting RAS components in preventing retinal vascular damage associated with HTN.

The study aimed to investigate the association between HR and serum and tear levels of RAS components (angiotensin II and ACE) in patients with primary hypertension with different degrees of HR, as well as to highlight the possible usefulness of these markers in the diagnosis of HR.

## Material and methods

### Study design and Participants

An analytical, observational study was conducted on a representative sample of patients diagnosed with primary hypertension and HR. The patients were enrolled at the Ovisus Ophthalmological Medical Center, Chişinău, Republic of Moldova. The sample size for the study was determined based on the estimated frequency of HR, which is reported to be approximately 17.0% based on recent literature data [9,26]. The patients were selected during primary random checks, and their diagnosis of HR was established through a comprehensive ophthalmological examination. The examination included assessments of visual acuity, auto refracto-keratometry, perimetry, anterior and fundus biomicroscopy (using the KWB grading system), ultrasonography, tonometry, gonioscopy, and optical coherence tomography.

A total of 90 hypertensive patients (mean age 59.79 ± 12.29 years; 52 females, 38 males) were grouped by the KWB grading system into Grade 1 (GI, n=36), Grade 2 (GII, n=35), and Grade 3 (GIII, n=19) HR. Patients with the 4th grade of HR were not included in our study due to a limited number of cases and the presence of other comorbidities [2,27].

### Inclusion and Exclusion Criteria

Patients were included if they had confirmed primary HTN and HR on fundoscopic assessment. Exclusion criteria comprised the use of antihypertensive drugs or other agents affecting biochemical markers, metabolic disorders (e.g., diabetes), severe obesity, advanced comorbidities, renal and neurological pathologies, ocular trauma, optic nerve atrophies, and ocular diseases such as DR or glaucoma.

### Sample collection

Venous blood (5 mL) was drawn and centrifuged at 3000 rpm for 5 minutes. Tear fluid was collected from the outer corner of the eyelid using a disposable insulin syringe, following mild irritation with Vietnamese “Golden Star” balm. Serum and tear samples were stored at -40°C.

### Biochemical analysis

Concentrations of Ang II and ACE in serum and tears were determined using standardized ELISA kits (Human Angiotensin II and ACE ELISA, MyBioSource, Inc., USA), according to the manufacturer’s protocols. Levels of Ang II and ACE were expressed in pg/mL.

### Statistical analysis

Data were analyzed in SPSS 28.0. Ang II and ACE levels were expressed as medians and interquartile ranges (IQR). Normality was tested by Kolmogorov-Smirnov and Shapiro-Wilk tests; Levene’s test assessed homogeneity of variance. Kruskal-Wallis and Mann-Whitney tests were used for multiple and pairwise group comparisons. Spearman correlation was employed to explore relationships between HR severity and the measured markers. A p-value ≤0.05 was considered significant.

### Ethical considerations

The study was approved by the Research Ethics Committee of the Nicolae Testemiţanu State University of Medicine and Pharmacy of the Republic of Moldova on February 12, 2018. Before participating in the study, all participants provided written informed consent.

## Results

A significant statistical enhancement in serum Ang II levels was observed as HR progressed (p=0.039). Conversely, there was a considerable diminishment in Ang II levels in tears (p=0.035).

**Fig. 1:**
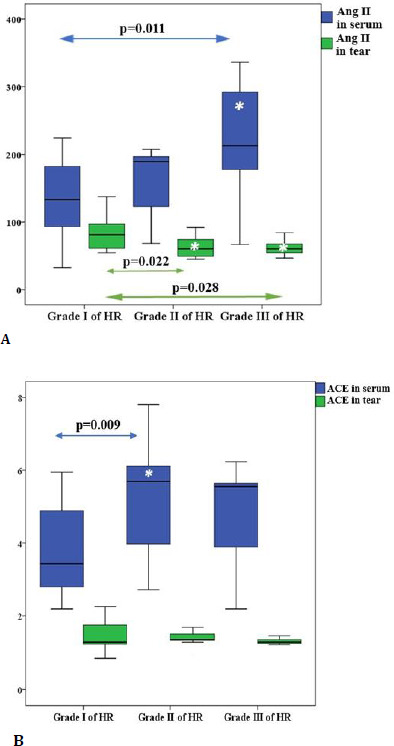
Levels of (**A**) Ang II (pg/mL) and (**B**) ACE (pg/mL) in serum and tears in patients with different grades of HR *Note:* Statistically significant difference compared to Group I of HR: * - p<0.05

When comparing the study groups, the serum Ang II level in GII showed a 42% increase compared to GI (189.64 (IQR 87.28) pg/mL vs. 133.17 (IQR 113.57) pg/mL, p=0.264), and a subsequent 18% augmentation in GIII compared to GII (213.01 (IQR 131.45) pg/mL vs. 189.64 (IQR 87.28) pg/mL, p=0.153) (**Fig. 1**). Additionally, a statistically significant difference was observed between GI (133.17 (IQR 113.57) pg/mL) and GIII (213.01 (IQR 131.45) pg/mL) with a p-value of 0.011.

In tears, a gradual decline in Ang II values was evident starting from GI (81.28 (IQR 41.01) pg/mL) to GII (60.48 (IQR 26.69) pg/mL) and GIII (60.48 (IQR 20.53) pg/mL), with a significant decrease of 26% between GI and GII (p=0.022), as well as between GI and GIII (p=0.028) (**Fig. 1**).

Significant negative sero-lacrimal correlations were found for Ang II (rs=-0.323, p=0.045), indicating a weak association. Furthermore, there was a statistically significant correlation between Ang II levels in both serum and tear and the severity of HR: the correlation was positive in serum (r_s_=0.413, p=0.009) and negative in tear (r_s_=-0.357, p=0.026), as shown in **Table 1**.

**Table 1:** Correlation between Ang II and ACE levels in serum/tear and the severity of HR

		Ang II		ACE	
		serum	tear	serum	tear
**Grade of retinopathy**	The correlation coefficient	0.413	- 0.357	0.236	-0.133
	Statistical significance, two-tailed test (p)	0.009	0.026	0.148	0.421

*Note:* Ang II = angiotensin II; ACE = angiotensin-converting enzyme.

At the same time, there was a statistically significant increase in the level of ACE in the blood serum (p=0.032), while no substantial changes in the tear level were observed (p=0.536).

In group comparisons, the serum level of ACE in GII showed a significant increase of 66% compared to GI (5.69 (IQR 2.60) pg/mL vs. 3.43 (IQR 2.44) pg/mL, p=0.009), while it remained at a similar level in GIII (5.55 (IQR 1.99) pg/mL vs. 5.69 (IQR 2.60) pg/mL, p=0.242) (**Fig. 1**).

Tear levels exhibited a statistically insignificant fluctuation, with a slight raise of 5% in GII (1.35 (IQR 0.20) pg/mL) compared to GI (1.28 (IQR 0.68) pg/mL, p>0.05), followed by a return to the initial values in GIII (1.28 (IQR 0.20) pg/mL) (**Fig. 1**).

The level of ACE in both serum and tear did not demonstrate a statistically significant correlation with the grade of retinopathy (**Table 1**).

## Discussion

Extensive research has revealed that HR is a complex, multifaceted condition that extends beyond elevated blood pressure, involving multiple mechanisms beyond HTN alone. Several reports suggest that local RASs regulate blood pressure and microcirculation more critically than systemic RAS, as nearly all RAS components are present in various ocular tissues [23,24]. While Ang II may be produced locally in the eye, its precise role remains under investigation [28-30]. Although RAS involvement has been explored in AMD and DR, its role in HR is largely uncharted [25,31-33].

In this study, serum Ang II rose significantly with advancing HR (p=0.039), with higher levels in Grades II and III, plus a negative correlation between serum and tear Ang II (r_s_=-0.323, p=0.045). Elevated systemic Ang II may drive vasoconstriction, vessel remodeling, inflammation, OS, and blood-retinal barrier disruption, as observed in other systems [12,22,24,25,33-35]. Meanwhile, tear Ang II decreased, possibly reflecting altered local production, tear composition changes, or a dilution effect [36]. This discrepancy merits further investigation.

Serum ACE rose significantly (p=0.032), aligning with the concept that sustained HTN activates RAS, enhancing Ang II production and exacerbating endothelial dysfunction [33]. Tear ACE levels and correlations with HR were not significant. Local ACE regulation may remain stable despite retinal damage or may be influenced by compensatory mechanisms [24,36,37]. The measurement of ACE in tears provides only a snapshot of ocular surface activity and does not necessarily reflect its function within the retina.

Overall, dysregulated local RAS seems critical in HR, potentially altering Ang II synthesis or degradation while preserving ACE. Varying adaptive responses across tissues may explain the discrepancy between serum and tear measurements. We analyzed patients not taking antihypertensive medications to avoid confounding effects. However, expanding research to include grade 4 HR, additional RAS components (e.g., renin, ACE2, AT1R/AT2R), and broader populations will help refine our understanding. Finally, this single-center study limits generalizability, underscoring the need for multi-center and cross-national investigations.

### Study limitations

This investigation was conducted at a single specialized medical center, potentially limiting generalizability. The relatively small sample size in higher-grade HR groups, particularly the lack of Grade 4, restricted analyses of late-stage retinopathy. We excluded hypertensive individuals on standard antihypertensive therapy to avoid confounding results. However, this prevents insight into how treatment might modulate Ang II and ACE levels. Finally, other RAS components (e.g., ACE2, aldosterone, or renin) could offer a more comprehensive view, and further multi-center, prospective studies are warranted.

## Conclusions

Our data highlighted that increasing serum Ang II levels and stable-to-elevated ACE values track with HR progression, suggesting that systemic RAS activation is linked to retinal vascular damage. Tear Ang II values decreased, likely reflecting altered local RAS regulation. These findings provide a rationale for future research into RAS-targeted strategies to mitigate retinal injury in hypertensive patients. Understanding the interplay between systemic and ocular RAS may inform novel biomarkers and therapeutic interventions for hypertensive retinopathy.

## References

[1] NCD Risk Factor Collaboration (NCD-RisC) (2021). Worldwide trends in hypertension prevalence and progress in treatment and control from 1990 to 2019: a pooled analysis of 1201 population-representative studies with 104 million participants. Lancet.

[2] Oparil S, Acelajado MC, Bakris GL, Berlowitz DR, Cífková R, Dominiczak AF, Grassi G, Jordan J, Poulter NR, Rodgers A, Whelton PK (2018). Hypertension. Nat Rev Dis Primers.

[3] Di Marco E, Aiello F, Lombardo M, Di Marino M, Missiroli F, Mancino R, Ricci F, Nucci C, Noce A, Di Daniele N, Cesareo M (2022). A literature review of hypertensive retinopathy: systemic correlations and new technologies. Eur Rev Med Pharmacol Sci.

[4] Cheung CY, Biousse V, Keane PA, Schiffrin EL, Wong TY (2022). Hypertensive eye disease. Nat Rev Dis Primers.

[5] Tan W, Yao X, Le TT, Tan B, Schmetterer L, Chua J (2022). The New Era of Retinal Imaging in Hypertensive Patients. Asia Pac J Ophthalmol (Phila).

[6] Kim DH, Chaves PH, Newman AB, Klein R, Sarnak MJ, Newton E, Strotmeyer ES, Burke GL, Lipsitz LA (2012). Retinal microvascular signs and disability in the Cardiovascular Health Study. Arch Ophthalmol.

[7] Pavlovschi E, Pantea V, Borovic D, Tagadiuc O (2021). Study of ischemia modified albumin (IMA) as a biomarker in hypertensive retinopathy. Med Pharm Rep.

[8] Pavlovschi E, Borovic D, Pantea V, Tagadiuc O (2021). Tear and serum superoxide dismutase and catalase activities in hypertensive retinopathy. Russ Open Med J.

[9] Ecaterina P, Valeriana P, Djina B, Olga T (2021). Glutathione-related antioxidant defense system in patients with hypertensive retinopathy. Rom J Ophthalmol.

[10] Pavlovschi E, Borovic D, Pantea V, Lîsîi L, Tagadiuc O (2021). Biomarkers of lipid status and metabolism in retinal hypertensive disorder. J Biosci Med.

[11] Pavlovschi E, Pantea V, Borovic D, Tagadiuc O (2021). Serum depletion and tear increase of total antioxidant capacity in hypertensive retinopathy. Arch Balk Med Union.

[12] Dziedziak J, Zaleska-Żmijewska A, Szaflik JP, Cudnoch-Jędrzejewska A (2022). Impact of Arterial Hypertension on the Eye: A Review of the Pathogenesis, Diagnostic Methods, and Treatment of Hypertensive Retinopathy. Med Sci Monit.

[13] Prakash D (2019). Target organ damage in newly detected hypertensive patients. J Family Med Prim Care.

[14] Kim DH, Newman AB, Hajjar I, Strotmeyer ES, Klein R, Newton E, Sarnak MJ, Burke GL, Lipsitz LA (2011). Retinal microvascular signs and functional loss in older persons: the cardiovascular health study. Stroke.

[15] Harjasouliha A, Raiji V, Garcia Gonzalez JM (2017). Review of hypertensive retinopathy. Dis Mon.

[16] Fraser-Bell S, Symes R, Vaze A (2017). Hypertensive eye disease: a review. Clin Exp Ophthalmol.

[17] Karaca M, Coban E, Felek R, Unal M (2013). The association of oxidative stress with hypertensive retinopathy. Clin Exp Hypertens.

[18] Karaca M, Coban E, Ozdem S, Unal M, Salim O, Yucel O (2014). The association between endothelial dysfunction and hypertensive retinopathy in essential hypertension. Med Sci Monit.

[19] Barthelmes J, Nägele MP, Ruschitzka F, Flammer AJ, Sudano I (2018). Eyes on hypertension: severe microvascular retinal dysfunction in hypertensive patients failing to achieve blood pressure treatment targets. Journal of Hypertension.

[20] Coban E, Nizam I, Topal C, Akar Y (2010). The association of low-grade systemic inflammation with hypertensive retinopathy. Clin Exp Hypertens.

[21] Coban E, Alkan E, Altuntas S, Akar Y (2010). Serum ferritin levels correlate with hypertensive retinopathy. Med Sci Monit.

[22] Sarlos S, Rizkalla B, Moravski CJ, Cao Z, Cooper ME, Wilkinson-Berka JL (2003). Retinal angiogenesis is mediated by an interaction between the angiotensin type 2 receptor, VEGF, and angiopoietin. Am J Pathol.

[23] Marin Garcia PJ, Marin-Castaño ME (2014). Angiotensin II-related hypertension and eye diseases. World J Cardiol.

[24] Choudhary R, Kapoor MS, Singh A, Bodakhe SH (2016). Therapeutic targets of renin-angiotensin system in ocular disorders. J Curr Ophthalmol.

[25] Holappa M, Vapaatalo H, Vaajanen A (2017). Many Faces of Renin-angiotensin System-Focus on Eye. Open Ophthalmol J.

[26] Modi P, Arsiwalla T Hypertensive Retinopathy 2025 Florida. https://www.ncbi.nlm.nih.gov/books/NBK525980/.

[27] Wong TY, Klein R, Sharrett AR, Duncan BB, Couper DJ, Klein BE, Hubbard LD, Nieto FJ, Atherosclerosis Risk in Communities Study (2004). Retinal arteriolar diameter and risk for hypertension. Ann Intern Med.

[28] Savaskan E, Löffler KU, Meier F, Müller-Spahn F, Flammer J, Meyer P (2004). Immunohistochemical localization of angiotensin-converting enzyme, angiotensin II and AT1 receptor in human ocular tissues. Ophthalmic Res.

[29] Senanayake PD, Drazba J, Shadrach K, Milsted A, Rungger-Brandle E, Nishiyama K, Miura S, Karnik S, Sears JE, Hollyfield JG (2007). Angiotensin II and its receptor subtypes in the human retina. Invest Ophthalmol Vis Sci.

[30] Wagner J, Jan Danser AH, Derkx FH, de Jong TV, Paul M, Mullins JJ, Schalekamp MA, Ganten D (1996). Demonstration of renin mRNA, angiotensinogen mRNA, and angiotensin-converting enzyme mRNA expression in the human eye: evidence for an intraocular renin-angiotensin system. Br J Ophthalmol.

[31] Klein R, Klein BE, Tomany SC, Cruickshanks KJ (2003). The association of cardiovascular disease with the long-term incidence of age-related maculopathy: the Beaver Dam Eye Study. Ophthalmology.

[32] Clermont A, Bursell SE, Feener EP (2006). Role of the angiotensin II type 1 receptor in the pathogenesis of diabetic retinopathy: effects of blood pressure control and beyond. J Hypertens Suppl.

[33] Wilkinson-Berka JL, Suphapimol V, Jerome JR, Deliyanti D, Allingham MJ (2019). Angiotensin II and aldosterone in retinal vasculopathy and inflammation. Exp Eye Res.

[34] Miura SI (2023). The renin-angiotensin-aldosterone system: a new look at an old system. Hypertens Res.

[35] Martyniak A, Tomasik PJ (2022). A New Perspective on the Renin-Angiotensin System. Diagnostics (Basel).

[36] Ravishankar P, Daily A (2022). Tears as the next diagnostic biofluid: a comparative study between ocular fluid and blood. Appl Sci.

[37] Abid A, Khan MA, Lee B, White A, Carnt N, Arshad S, Samarawickrama C (2022). Ocular Distribution of the Renin-Angiotensin-Aldosterone System in the Context of the SARS-CoV-2 Pandemic. J Renin Angiotensin Aldosterone Syst.

